# Hes-1 SUMOylation by protein inhibitor of activated STAT1 enhances the suppressing effect of Hes-1 on GADD45α expression to increase cell survival

**DOI:** 10.1186/1423-0127-21-53

**Published:** 2014-06-04

**Authors:** Hsin-Ying Clair Chiou, Shau-Yu Liu, Cheng-Hsiung Lin, Eminy HY Lee

**Affiliations:** 1 Graduate Institute of Life Sciences, National Defense Medical Center, Taipei, Taiwan; 2 Institute of Biomedical Science, Academia Sinica, Taipei 115, Taiwan

**Keywords:** Hes-1, PIAS1, GADD45α, SUMOylation, Cell survival

## Abstract

**Background:**

*Hairy* and *Enhancer of split 1* (Hes-1) is a transcriptional repressor that plays an important role in neuronal differentiation and development, but post-translational modifications of Hes-1 are much less known. In the present study, we aimed to investigate whether Hes-1 could be SUMO-modified and identify the candidate SUMO acceptors on Hes-1. We also wished to examine the role of the SUMO E3 ligase protein inhibitor of activated STAT1 (PIAS1) in SUMOylation of Hes-1 and the molecular mechanism of Hes-1 SUMOylation. Further, we aimed to identify the molecular target of Hes-1 and examine how Hes-1 SUMOylation affects its molecular target to affect cell survival.

**Results:**

In this study, by using HEK293T cells, we have found that Hes-1 could be SUMO-modified and Hes-1 SUMOylation was greatly enhanced by the SUMO E3 ligase PIAS1 at Lys8, Lys27 and Lys39. Furthermore, Hes-1 SUMOylation stabilized the Hes-1 protein and increased the transcriptional suppressing activity of Hes-1 on growth arrest and DNA damage-inducible protein alpha (GADD45α) expression. Overexpression of GADD45α increased, whereas knockdown of GADD45αα expression decreased cell apoptosis. In addition, H_2_O_2_ treatment increased the association between PIAS1 and Hes-1 and enhanced the SUMOylation of Hes-1 for endogenous protection. Overexpression of Hes-1 decreased H_2_O_2_-induced cell death, but this effect was blocked by transfection of the Hes-1 triple sumo-mutant (Hes-1 3KR). Overexpression of PIAS1 further facilitated the anti-apoptotic effect of Hes-1. Moreover, Hes-1 SUMOylation was independent of Hes-1 phosphorylation and *vice versa*.

**Conclusions:**

The present results revealed, for the first time, that Hes-1 could be SUMO-modified by PIAS1 and GADD45α is a novel target of Hes-1. Further, Hes-1 SUMOylation mediates cell survival through enhanced suppression of GADD45α expression. These results revealed a novel role of Hes-1 in addition to its involvement in Notch signaling. They also implicate that SUMOylation could be an important posttranslational modification that regulates cell survival.

## Background

*Hairy* and *Enhancer of split 1* (Hes-1) is a transcriptional repressor belongs to the basic helix-loop-helix (bHLH) protein family, and was shown to play a pivotal role in regulation of cell differentiation and proliferation in various cell types during development [1]. Hes-1 is a Notch effector and can repress the transcription of its target genes through sequestration of other transcription activators or recruitment of cofactors [2]. Through forming homodimers, Hes-1 directly binds to the N-box (CACNAG) of target gene promoter and recruits transducin-like enhancer to repress transcription. Hes-1 also forms heterodimers with other bHLH activators and sequesters them from binding to the E-box (CANNTG) of target gene promoter and that results in passive repression.

The repression activity of Hes-1 can be regulated by protein phosphorylation. Our recent finding indicates that phosphorylation of Hes-1 at Ser263 by c-Jun N-terminal kinase 1 (JNK1) stabilizes the Hes-1 protein and enhances its suppressing effect on α-amino-3-hydroxy-5-methyl-4-isoxazolepropionic acid receptor subunit GluR1 expression [3]. Moreover, phosphorylation at protein kinase C consensus sites (Ser37, Ser38) in the basic domain of Hes-1 inhibits the DNA-binding activity of Hes-1 during nerve growth factor stimulation of PC12 cell differentiation [4]. In addition, Hes-1 phosphorylation by calmodulin-dependent protein kinase II delta turns it from a repressor to an activator that is required for neuronal stem cell differentiation [5]. But in addition to Hes-1 phosphorylation, whether other posttranslational modification also occurs to Hes-1 is barely known.

Post-translational modification of proteins with small ubiquitin-like modifier (SUMO) has been recognized as an important mechanism for regulation of various cellular functions [6]. SUMO is a polypeptide about 100 amino acids in length that is covalently attached to substrate proteins on the lysine (Lys) residue. In the SUMO pathway, SUMO precursors are first processed by SUMO-specific proteases and activated by E1 enzyme, and subsequently transferred to the E2 conjugation enzyme UBC9. The SUMO E3 ligases then transfer the SUMO molecule from UBC9 to specific substrate proteins [7]. Protein inhibitor of activated STAT1 (PIAS1) is a SUMO E3 ligase belongs to the PIAS protein family that is well studied in the immune system [8,9]. Through ligase activity-dependent or -independent mechanism, PIAS1 regulates the activity of distinct proteins, including transcription factors [10]. For example, we have previously shown that PIAS1 facilitates spatial learning and memory in rats through enhanced SUMOylation of STAT1 and decreased phosphorylation of STAT1 [11]. Further, PIAS1 promotes the SUMOylation of mastermind-like 1 (MAML1), a co-activator of NICD, and enhances its association with histone deacetylase 7 and decreases the transcriptional activity of MAML1 [12]. The latter results indicate that PIAS1 could modulate Notch signaling through SUMOylation of different transcriptional co-repressors or co-activators of the Notch signaling pathway. In the present study, we examined whether PIAS1 could modulate the activity of the Notch effector Hes-1 through SUMOylation of Hes-1. We also studied the molecular mechanism and cellular function of Hes-1 SUMOylation.

## Methods

### Drugs

Cycloheximide and N-ethylmaleimide (NEM) were purchased from Sigma-Aldrich (St. Louis, MO, USA). Calf intestinal phosphatase (CIP) was purchased from NEB (Ipswich, MA, USA).

### *In vitro* SUMOylation assay

*In vitro* sumoylation assay was performed using the SUMO link™ kit according to the manufacturer’s instructions (Active Motif, Carlsbad, CA). Briefly, purified recombinant proteins were mixed and incubated at 30°C for 4 h, and the reaction was stopped by boiling in Laemmli sample buffer at 95°C for 10 min. The product was analyzed by 10% SDS-PAGE then transferred onto the PVDF membrane (Millipore, Bedford, MA). The membrane was immunoblotted with antibodies against Hes-1 (GeneTex, Irvine, CA) and SUMO-1 (Active Motif).

### Plasmid DNA construction

For construction of the Flag-tagged *pias1* plasmid, full-length *pias1* was cloned by amplifying the rat *pias1* cDNA. The PCR product was subcloned between the *BamHI* and *EcoRI* sites of the expression vector pCMV-Tag2A. Flag-tagged *pias2*, *pias3* and *pias4* plasmids were prepared in the same way. The PCR products were subcloned between the *EcoRI* and *XhoI* sites of the expression vector pCMV-Tag2A. For construction of the EGFP-tagged *pias1* plasmid, full-length *pias1* was subcloned into the pEGFP-C1 expression vector with RsrII site. For construction of the Flag-tagged *Hes-1* plasmid, full-length *Hes-1* was cloned by amplifying the rat *Hes-1* cDNA. The PCR product was subcloned between the *BamHI* and *EcoRI* sites of the expression vector pCMV-Tag2B. For construction of the Flag-tagged *Hes-5* plasmid, full-length *Hes-5* was cloned by amplifying the rat *Hes-5* cDNA. The PCR product was subcloned between the *BamHI* and *EcoRI* sites of the expression vector pCMV-Tag2B. For construction of the Flag-tagged *RanBP2(ΔFG)* plasmid, partial-length *RanBP2* (amino acid 2553-2838) was cloned by amplifying the human kidney *RanBP2* cDNA. The PCR product was subcloned between the *EcoRI* and *XhoI* sites of the expression vector pCMV-Tag2B. For construction of the Flag-tagged *Pc2* plasmid, full-length *Pc2* was cloned by amplifying the rat *Pc2 c*DNA. The PCR product was subcloned between the *EcoRI* and *XhoI* sites of the expression vector pCMV-Tag2A. For construction of the Myc-tagged *sumo-1* plasmid, full-length *sumo-1* was cloned by amplifying the mouse *sumo-1 c*DNA. The PCR product was subcloned between the *XmaI* and *KpnI* sites of the expression vector pCMV-Myc. For construction of the His-tagged *ubiquitin* plasmid, full-length *ubiquitin* was cloned by amplifying the human *ubiquitin c*DNA. The PCR product was subcloned between the *NcoI* and *BamHI* sites of the expression vector CMV-3 × His tag vector. The *pias1S90A* and *pias1W372A* mutant plasmids, *Hes-1* phosphorylation mutants and sumo-mutant plasmids were generated by using the QuickChange Site-Directed Mutagenesis Kit (Stratagene, La Jolla, CA). For construction of the human GADD45α promoter-luciferase fusion plasmid (pGL3-GADD45α -P), nt.-920 ~ nt. + 289 of GADD45α promoter was inserted into the pGL3 basic vector (Promega, Madison, WI) between SacI and BglII restriction enzyme sites. The nt.-920 ~ nt. + 289 of GADD45α promoter region was generated by PCR amplification from human genomic DNA. The primer sequences used for the above plasmid cDNAs are summarized in Table 1.

**Table 1:** Primers for plasmid construction

Pias1	Forward: 5’-ATCGGGATCCCATGGCGGACAGTGCGGAAC-3’
	Reverse: 5’-ATCGGAATTCTCAGTCCAACGAGATAATG-3’
Pias2	Forward: 5’-ATCGGAATTCGATGGCGGATTTCGAGGAG-3’
	Reverse: 5’-ATCGCTCGAGTCACTGTTGCACAGTATC-3’
Pias3	Forward: 5’-ATCGGAATTCGATGGCGGAGCTGGGCG-3’
	Reverse: 5’-ATCGCTCGAGTCAGTCCAAGGAAATG-3’
Pias4	Forward: 5’-ATCGGAATTCGATGGCGGCAGAGCTGGTG-3’
	Reverse: 5’-ATCGCTCGAGTCAGCAAGCGGGCACCAG-3’
Hes-1	Forward: 5’-ATGCCAGCTGATATAATGG-3
	Reverse: 5’-TCAGTTCCGCCACGGCCTC-3
Hes-5	Forward: 5’-GACTGGATCCATGGCCCCAAGTACCGTGG-3’
	Reverse: 5’-GACTGAATTCTCACCAGGGCCGCCAGAG-3’
RanBP2	Forward: 5’-ATGCGAATTCTTGAAAAGTAACAATAG-3’
	Reverse: 5’-ATGCCTCGAGAACTATCTTGCTTTCC-3’
Pc2	Forward: 5’-ATCGGAATTCGATGGAGCTGCCAGCTGTTG-3’
	Reverse: 5’-ATCGCTCGAGCTACACCGTCACGTATTC-3’
SUMO1	Forward: 5’-GCAACCCGGGTGTCTGACCAGGAGGCAAAACCTTC-3’
	Reverse: 5’-GCAAGGTACCCTAAACCGTCGAGTGACCCCCCGT-3’
Ubiquitin	Forward: 5’-CCATGGATGCAGATCTTCGTGAAGAC-3’
	Reverse: 5’-GGATCCTTAGACACCCCCCCTCAAGC-3’
GADD45α	Forward: 5’-GACTGAGCTCCTTAGGGCATATCGAGAGCAT-3’
	Reverse: 5’-GATCAGATCTAAAGTCATATTGCAAACTGCAGGTC-3’

### RNA interference

Various siRNAs were used to knock down the expression of individual proteins specifically. For PIAS1 and Hes-1, two sets of different siRNAs were used. The sense and antisense sequences for all the siRNA used are summarized in Table 2. The Silencer Negative Control number 1 siRNA (control siRNA) was used as a control. These are the siRNAs with sequences that do not target any gene product (Ambion, Austin, TX).

**Table 2:** siRNA sequences

Pias1 #1	sense: 5’-AGAAAUGUACAGAGAACAAdTdT -3’
	antisense: 5’-UUGUUCUCU GUACAUUUCUdTdT -3’
Pias1 #2	sense: 5’- GAACUAAAGCAAAUGGUUAdTdT -3’
	antisense: 5’-UAACCAUUU GCUUUAGUUCdTdT-3’
Pias2	sense: 5’-GAUACUAAGCCCACAUUUGdTdT-3’
	antisense: 5’-CAAAUGUGGGCUUAGUAUCdTdT-3’
Pias3	sense: 5’-CCCUGAUGUCACCAUGAAAdTdT-3’
	antisense: 5’-UUUCAUGGUGACAUCAGGGdTdT-3’
Hes-1 #1	sense: 5’-CCACGUGCGAGGGCGUUAAdTdT-3’
	antisense: 5’-UUAACGCCCUCGCACGUGGdTdT-3’
Hes-1 #2	Target 1: 5’-CGAAGAGCAAGAAUAAAUG-3’
(Thermo)	Target 2: 5’-UGAACGAGGUGACCCGCUU-3’
	Target 3: 5’-AGAUCAAUGCCAUGACCUA-3’
	Target 4: 5’-GAAGAAAGAUAGCUCGCGG-3’
GADD45α	sense: 5’-GGAUCCUGCCUUAAGUCAACUUAdTdT-3’
	antisense: 5’-UAAGUUGACUUAAGGCAGGAUCCdTdT-3’

### Cell culture and plasmid transfection

HEK293T cells were grown in DMEM (Hyclone, Logan, UT, USA) supplemented with 10% fetal bovine serum (Hyclone) in a humidified atmosphere at 5% CO_2_ at 37°C. Cells were transfected with various plasmids by Lipofectamin 2000 24 h after seeding (Invitrogen, Carlsbad, CA) according to the manufacturer’s protocol. Briefly, Lipofectamine 2000 was pre-mixed with 100 μl of DMEM at a ratio of DNA: Lipofectamine = 1 μg: 2.5 μl for 5 min at room temperature. Plasmid DNA was resolved in 100 μl DMEM and mixed with the pre-mixed Lipofectamine. After incubation for 20 min at room temperature, the mixture was added to cells in culture medium without antibiotics.

### Immunoprecipitation (IP) assay

Cells transfected with plasmid DNA were lysed in RIPA buffer with the addition of protease inhibitor and phosphatase inhibitor (Roche). The cell extracts were harvested by centrifugation at 4°C for 10 min and the supernatant was used for IP assay. Cell extracts (500 μg) were incubated with 20 μl of anti-Flag M2 affinity gel (50% slurry) (Sigma-Aldrich, St. Louis, MO) at 4°C for 2 h. Immunoprecipitates were collected by centrifugation at 1000 × g, washed with RIPA buffer for three times followed by SDS-PAGE electrophoresis and western blot. For co-IP assay, cells were co-transfected with equal amount of Tag-fusioned constructs and lysed 48 h later. Anti-Flag M2 affinity gel (50% slurry) (Sigma-Aldrich) and EGFP antibody (Roche) were used for IP of protein complex from cell extract at 4°C for 2 h. After washing with PBS for three times, the immunoprecipitates were eluted by sampling buffer and subject to SDS-PAGE and western blot.

### Western blot

HEK293T cells were lysed in RIPA buffer [50 mM Tris-HCl (pH 7.4), 150 mM NaCl, 2 mM EDTA, 1% IGEPAL CA-630, 1 mM phenymethylsulfonyl fluoride (PMSF), 20 mg/ml pepstatin A, 20 mg/ml leupeptin, 20 mg/ml aprotinin, 50 mM NaF and 1 mM Na_3_VO_4_] and the protein lysates were harvested by centrifugation at 14,000 rpm to remove the debris. The lysate was resolved by 10% or 12% SDS-PAGE and proteins separated by SDS-PAGE were transferred onto the PVDF membrane for antibody conjugation. The antibodies used include: anti-Flag M2 (Sigma-Aldrich, St. Louis, MO), anti-actin (Millipore), anti-EGFP (Roche, Penzberg, Germany), anti-GADD45α (Santa Cruz Biotechnology, Santa Cruz, CA), anti-SUMO-1 (Active Motif), anti-SUMO-2 (Epitomics, Burlingame, CA), anti-Hes-1 (GeneTex), anti-pSer263 Hes-1 [3], anti-PIAS1 (Epitomics), anti-pSer90PIAS1 (LTK BioLaboratories, Taoyuan, Taiwan), anti-α-His (Millipore) and anti-Myc (Millipore) antibodies. The secondary antibodies used were HRP-conjugated goat-anti-mouse IgG antibody and HRP-conjugated goat-anti-rabbit IgG antibody (Jackson ImmunoResearch Laboratories, West Grove, PA). Membrane was developed by reacting with chemiluminescence HRP substrate (Millipore) and exposed to the LAS-3000 image system (Fujifilm, Tokyo, Japan) for visualization of protein bands.

### Promoter activity assay

Cells were plated at a density of 5 × 10^4^ cells/well and transfected with 0.4 μg of pGL3-GADD45α-P promoter-firefly luciferase reporter plasmid, 0.001 μg pRL (Rellina) and 1.2 μg of various plasmid DNA 24 h later. The total mass of transfected DNA in each well was kept constant by adding empty vector plasmid DNA when necessary. Forty-eight hours after transfection, cells were washed with PBS and lysed with 1× Passive Lysis Buffer (Promega). Luciferase activity was determined using the Dual-Glo luciferase assay system (Promega) and the TD-20/20 Luminometer (Turner Designs Hydrocarbon Instruments). The relative activity was normalized to the Rellina activity.

### Quantitative real-time PCR

Total RNA was isolated using the RNAspin mini kit (GE Healthcare, Buckinghamshire, Germany). Purified RNA (1 μg) was reverse-transcripted to cDNA by SuperScript III reverse transcriptase (Invitrogen). Quantitative real-time PCR was performed using the ABI PRISM 7500 real-time PCR system with Power SYBR Green PCR reagents (Fermentas, Vilnius, Lithuania) according to the instruction manual (Applied Biosystem, ABI, Foster City, CA). The primer sequences for GADD45α are as follows: 5′-GAGAGCAGAAGACCGAAAGGA-3′ (forward) and 5′-CACAACACCACGTTATCGGG-3′ (reverse). HPRT was used as an internal control for each sample. The primer sequences for HPRT are: 5′-TGTGTGCTCAAGGGGGGC-3′ (forward) and 5′-CGTGGGGTCCTTTTCACC-3′ (reverse). The amount of *gadd45*α gene expression is normalized to that of *HPRT* gene expression.

### Chromatin immunoprecipitation (ChIP) assay

ChIP was performed according to the manufacturer’s protocol (Millipore). Briefly, nuclear chromatin extracts were incubated with antibody against Hes-1 (Novus, Littleton, CO) (4.8 μg of anti-Hes-1 or mouse IgG) or Flag-M2 (Sigma-Aldrich) at 4°C overnight. Immunoprecipitates were collected on magnesium beads for another 1-2 h at 4°C. After thorough washing, immunoprecipitates were de-crosslinked and chromatin was recovered for quantitative PCR analysis. Primers used for GADD45α promoter are: 5′-TCATGATTCAGCATCTAACATCAATAA-3′ (forward) and 5′-GACAACCATCTGACACCC-3′ (reverse).

### Immunofluorescence staining

Immunofluorescence staining was performed as described previously [3]. HEK293T cells were fixed with 4% paraformaldehyde/4% sucrose for 10 min followed by permeabilization with 0.1% Triton X-100 for 20 min at room temperature. Primary antibody against Hes-1 (GeneTex) (1:100) was added to the cells with 0.5% bovine serum albumin at 4°C overnight. Cy5 donkey anti-rabbit antibody (Jackson ImmunoResearch Laboratories) (1:500) was incubated with cells for 1 h at room temperature. After washed with PBS, cells were stained with DAPI and examined under a fluorescence microscope.

### Cytotoxicity assay

The cytotoxicity of H_2_O_2_ on HEK293T cells was determined by using CCK-8 assay (Cell Counting Kit, Boster Biological Technology, Ltd., Fremont, CA) [13]. Cells were seeded in 96-well plate at a density of 4 × 10^3^ cells/well in 100 μl of culture medium. After incubation for 24 h, the cells were transfected with various plasmids by Lipofectamine 2000. Forty-eight hours after transfection, cells were treated with different concentrations of H_2_O_2_ for 4 h. Next, 10 μl (1/10 v/v) of CCK-8 reagent was added to each well and incubated for another 2 h in incubator. Wells containing no cell (medium only) but treated with CCK-8 were used as the blank control, and cells transfected with vector plasmid and CCK-8 but no H_2_O_2_ challenge served as the negative control. After incubation, cell viability was determined by using the microplate reader (SpectraMax340pc384, Molecular Devices, Sunnyvale, CA) with absorbance set at 450 nm. The absorbance reading from each well was used to calculate the cell survival rate. Survival rate(%) = [optical density(OD)of the treated cells - OD of blank control/OD of negative control - OD of blank control] × 100(%).

### Terminal deoxynucleotidyl transferase dUTP nick end labeling (TUNEL) assay

HEK293T cells were plated in 24-well plates at a density of 2 × 10^4^ cells/well. Twenty-four hours after plating, cells were transfected with plasmid DNA or siRNA. Forty-eight hours after transfection, cells were treated with different concentrations of H_2_O_2_ and subject to TUNEL assay. TUNEL assay was performed using Apoptag plus peroxidase *in situ* apoptosis detection kit (Millipore). Briefly, after H_2_O_2_ treatment, cells were fixed in 1% paraformaldehyde for 10 min at room temperature and post-fixed by EtOH/CH_3_COOH (2:1) for 5 min at -20°C. Cells were then incubated with TdT enzyme for 1 h at 37°C followed by incubation with anti-digoxigenin peroxidase for 30 min at room temperature. The apoptotic cells yield brown color after DAB staining viewed from a light microscope.

### Statistics

Data are analyzed by Student’s t-test (for two groups) or one-way analysis of variance followed by Newman-Keul multiple comparisons (for more than two groups). Statistically significant levels are tested at *p* < 0.05, *p* < 0.01 and *p* < 0.001.

## Results

### Hes-1 is a SUMO substrate of PIAS1

**Figure 1:**
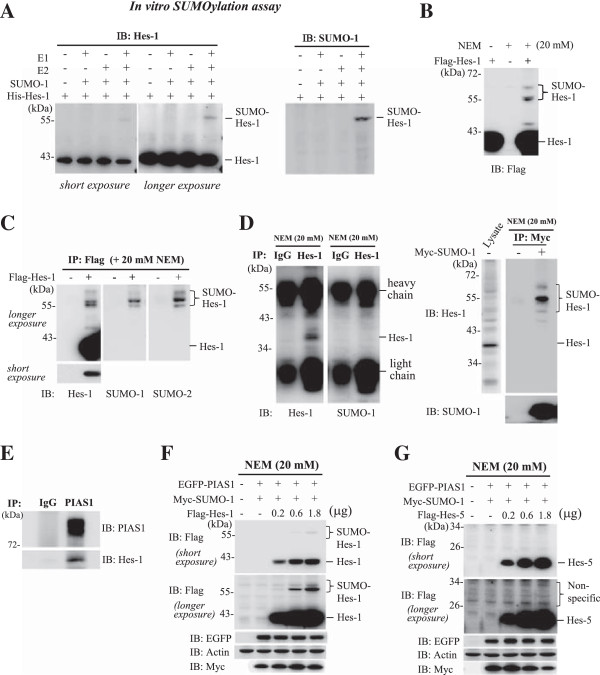
**Hes-1 is sumoylated both *in vitro *and *in vivo*. (A)** Recombinant Hes-1 protein was purified from *E. coli*. and *in vitro* SUMOylation assay was carried out in the presence (or absence) of E1, E2, and SUMO-1 followed by western blot against Hes-1 (left panel) or SUMO-1 (right panel). **(B)** Flag-Hes-1 WT plasmid was transfected to HEK293T cells and the cell lysate was subject to western blot against Flag. **(C)** HEK293T cells were transfected with Flag-vector or Flag-Hes-1WT plasmid and the cell lysate was subject to immunoprecipitation with anti-Flag antibody and immunoblotting with antibody against Hes-1 (left), SUMO-1 (middle) or SUMO-2 (right). **(D)** HEK293T cells were lysed and immunoprecipitated with anti-Hes-1 (or IgG) antibody and immunoblotted with anti-Hes-1 (left) or anti-SUMO-1 (right) antibody (left panel). Myc-SUMO-1 plasmid was transfected to HEK293T cells and the cell lysate was immunoprecipitated with anti-Myc antibody and immunoblotted with anti-Hes-1 or anti-SUMO-1 antibody (right panel). **(E)** HEK293T cell lysate was immunoprecipitated with anti-PIAS1 antibody and immunoblotted with anti-Hes-1 or anti-PIAS1 antibody. **(F)** EGFP-PIAS1 plasmid, Myc-SUMO-1 plasmid and different doses of Flag-Hes-1 plasmid were transfected to HEK293T cells and the cell lysates were subject to western blot using antibodies against Flag, EGFP and Myc. **(G)** EGFP-PIAS1 plasmid, Myc-SUMO-1 plasmid and different doses of Flag-Hes-5 plasmid were transfected to HEK293T cells and the cell lysates were subject to western blot using antibodies against Flag, EGFP and Myc. The sumoylated Hes-1 bands are shown in the bracket. Each experiment was performed twice.

To examine whether Hes-1 could be SUMO-modified, recombinant Hes-1 protein was subject to *in vitro* SUMOylation assay and western blot. Results showed that a band around 55 kDa was observed when E1, E2, SUMO-1 and Hes-1 proteins were added to the reaction (Figure 1A, left panel). The ratio of sumoylated Hes-1 versus un-sumoylated Hes-1 is approximately 8%. This band was also recognized by the SUMO-1 antibody (Figure 1A, right panel), indicating that Hes-1 can be sumoylated *in vitro*. Next, Flag-Hes-1 plasmid was transfected to HEK293T cells with or without the addition of NEM, a potent SUMO protease inhibitor [14]. Immunoblotting using anti-Flag antibody resolved a major band around 40 kDa in all lysates, but three slowly migrating bands near 55 kDa were also observed in cell lysates treated with NEM (Figure 1B), indicating that these bands may be the sumoylated form of Hes-1. The reason for observation of three migrating bands is probably because poly-sumoylation occurs to Hes-1 that makes the molecular weight of sumoylated Hes-1 different. In addition, the slowly migrating band just above the 43 kDa of Hes-1 is probably the phosphorylated Hes-1 because we have later found that treatment of the phosphatase CIP prevented the observation of this band. To confirm that both SUMO-1 and SUMO-2 can be conjugated to Hes-1, Flag-Hes-1 plasmid was transfected to HEK293T cells and cells were immunoprecipitated with anti-Flag antibody followed by immunoblotting with different antibodies. Results revealed that the slowly migrating bands were also observed when immunoblotted with the anti-Hes-1, anti-SUMO-1 or anti-SUMO-2 antibody (Figure 1C). Next, we examined whether endogenous Hes-1 could be sumo-modified. HEK293T cell lysates were directly immunoprecipitated with anti-Hes-1 antibody (or IgG) and immunoblotted with anti-Hes-1 and anti-SUMO-1 antibodies. Results showed that under this condition, the SUMO-Hes-1 bands were interfered by the heavy chain (Figure 1D, left panel). We therefore transfected the Myc-SUMO-1 plasmid to HEK293T cells (without co-transfection of Hes-1 plasmid) and the cell lysates were immunoprecipitated with anti-Myc antibody and immunoblotted with anti-Hes-1 and anti-SUMO-1 antibodies. Results revealed that endogenous Hes-1 SUMOylation could be observed under this situation (Figure 1D, right panel). We then examined whether PIAS1 is associated with Hes-1 endogenously. The HEK293T cell lysates were directly immunoprecipitated with anti-PIAS1 antibody without any plasmid transfection and immunoblotted with anti-Hes-1 and anti-PIAS1 antibody. Result revealed that PIAS1 is associated with Hes-1 endogenously (Figure 1E). However, because we aimed to examine the effects of Hes-1WT and Hes-1 sumo-mutants on cell survival in the present study, but the Hes-1 sumo-mutant proteins are not present in the cells endogenously, we have therefore adopted the overexpression strategy for the subsequent experiments. We also examined the possible SUMOylation of Hes-5, another member of the Hes protein family. Results revealed a dose-dependent increase in Hes-1 SUMOylation upon Hes-1 plasmid transfection to HEK293T cells (Figure 1F), but none of these doses of Hes-5 plasmid transfection resulted in Hes-5 SUMOylation (Figure 1G).

### PIAS1 is associated with Hes-1 and enhances the SUMOylation of Hes-1

**Figure 2:**
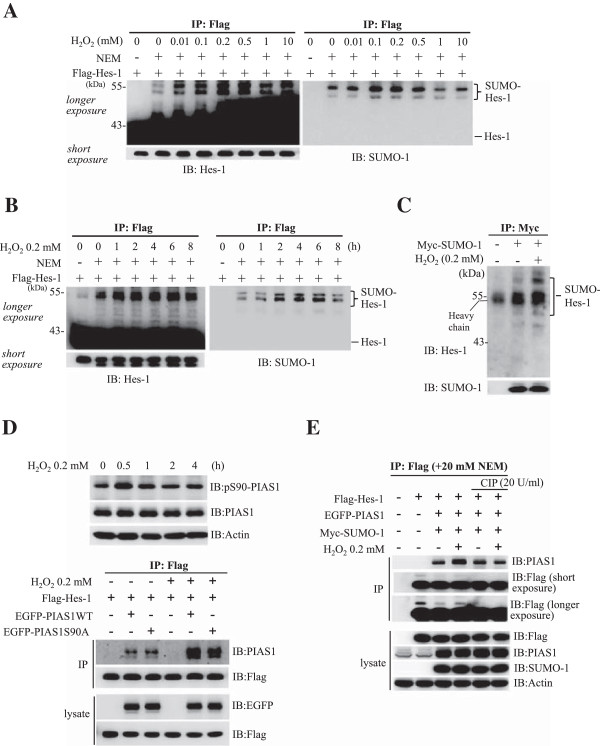
**H**_**2**_**O**_**2 **_**enhances the SUMOylation of Hes-1. (A)** Flag-Hes-1WT plasmid was transfected to HEK293T cells with the addition of different concentrations of H_2_O_2_ and the cell lysate was immunoprecipitated with anti-Flag antibody and immunoblotted with anti-Hes-1 (left) or anti-SUMO-1 (right) antibody. **(B)** Flag-Hes-1WT plasmid was transfected to HEK293T cells with the addition of 0.2 mM H_2_O_2_ for different time periods. The cell lysate was immunoprecipitated with anti-Flag antibody and immunoblotted with anti-Hes-1 (left) or anti-SUMO-1 (right) antibody. **(C)** Myc-SUMO-1 plasmid was transfected to HEK293T cells with the addition of 0.2 mM H_2_O_2_ for 4 h. The cell lysate was immunoprecipitated with anti-Myc antibody and immunoblotted with anti-Hes-1 and anti-SUMO-1 antibody. **(D)** H_2_O_2_ (0.2 mM) was added to HEK293T cells for different time periods and the cell lysate was subject to western blot against pSer-90 PIAS1 and PIAS1 (upper panel). Flag-Hes-1WT plasmid was transfected to HEK293T cells with co-transfection of EGFP-PIAS1WT or EGFP-PIAS1S90A plasmid and with the addition of H_2_O_2_ for 4 h and the cell lysate was immunoprecipitated with anti-Flag antibody and immunoblotted with antibodies against PIAS1 and Flag. Western blot against EGFP and Flag for cell lysates only was used as a control for transfection and expression (lower panel). **(E)** Flag-Hes-1WT plasmid was co-transfected with EGFP-PIAS1 and Myc-SUMO-1 plasmids, and H_2_O_2_ was added to some of these groups for 4 h. The phosphatase inhibitor CIP (20 U/ml) was also added to some of these groups. The cell lysates were immunoprecipitated with anti-Flag antibody and immunoblotted with anti-PIAS1 antibody. Western blot against Flag, PIAS1 and SUMO-1 for cell lysates only was used as loading controls. The sumoylated Hes-1 bands are shown in the bracket. Each experiment was performed twice.

The above results indicated that *in vivo* Hes-1 SUMOylation is more apparent than that observed *in vitro*, it suggests that other components in the SUMO machinery contribute to Hes-1 SUMOylation in the cell, particularly the E3 ligase, which is known to promote the SUMOylation reaction and enhance substrate specificity [7]. There are three families of the SUMO E3 ligase including PIAS [15], RanBP2 [16] and Pc2 [17]. Furthermore, the PIAS family consists of different members of the PIAS protein, including PIAS1, PIAS2, PIAS3 and PIAS4 [18]. Here, we examined whether these E3 ligases enhance the SUMOylation of Hes-1. EGFP-Hes-1 and Myc-SUMO-1 were co-transfected with Flag-RanBP2 (ΔFG), Flag-Pc2 or individual Flag-PIAS plasmid (PIAS1 to PIAS4) to HEK293T cells and subject to western blot. Results revealed that Hes-1 SUMOylation could be enhanced by PIAS1, PIAS2 and PIAS3, but not by PIAS4 and RanBP2 (ΔFG) (Figure 2A). Overexpression of Pc2 at different doses did not promote the SUMOylation of Hes-1 either (Figure 2B). To further examine the role of PIAS1, PIAS2 and PIAS3 in Hes-1 SUMOylation, we have transfected siRNA against PIAS1, PIAS2 and PIAS3 to HEK293T cells, respectively and have found that, consistent with the result of overexpression, PIAS1 siRNA and PIAS3 siRNA more apparently decreased the SUMOylation of Hes-1 (Figure 2C).

Both PIAS proteins and Hes-1 are suggested to locate in the nucleus [19,20]. Next, we examined the sub-cellular localization of PIAS1 and Hes-1 in HEK293T cells by immunofluorescence staining. DAPI was used as a nuclear marker. Results revealed that both PIAS1 and Hes-1 showed nuclear localization and the merged image indicated that PIAS1 is co-localized with Hes-1 (Figure 2D). Co-immunoprecipitation assay also showed a physical association between PIAS1 and Hes-1 in the cell (Figure 2E). We then determined whether Hes-1 SUMOylation by PIAS1 depends on the ligase activity of PIAS1. PIAS1W372A was used for this purpose because Trp372 of PIAS1 is located in the SP-RING domain which is important for the SUMO reaction (Figure 2F, upper panel), and mutation of this Trp residue to Ala was shown to lose PIAS1 E3 ligase activity [21]. Various combinations of Flag-Hes-1, Myc-SUMO-1, EGFP-PIAS1WT or EGFP-PIAS1W372A plasmids were transfected to HEK293T cells for immunoblot. Results revealed that PIAS1WT transfection apparently promotes the SUMOylation of Hes-1, but PIAS1W372A transfection completely blocked this effect (Figure 2F, lower panel).

### H_2_O_2_ enhances the SUMOylation of Hes-1

**Figure 3:**
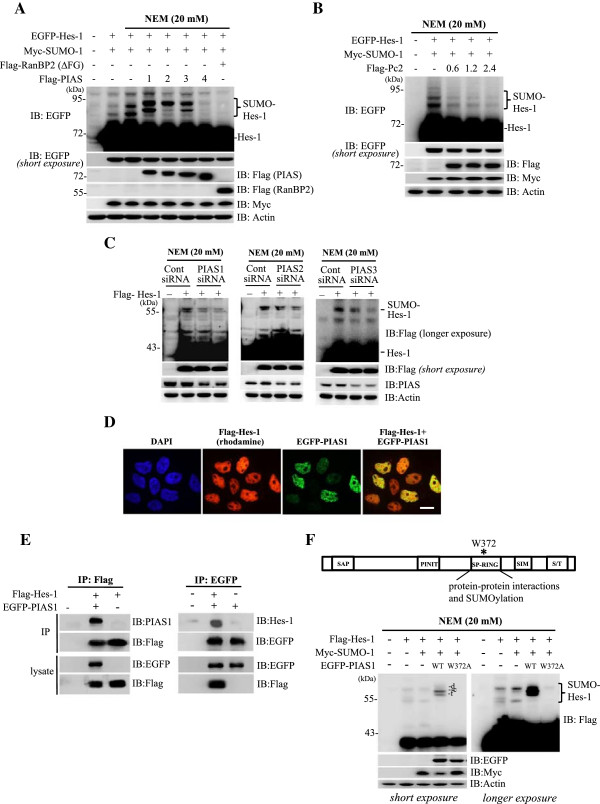
**PIAS1 is associated with Hes-1 and PIAS1 enhances the SUMOylation of Hes-1 in HEK293T cell. (A)** Transfection of various plasmids to HEK293T cells followed by western blots against EGFP and various tags. **(B)** EGFP-Hes-1, Myc-SUMO-1 and different doses of Flag-Pc2 plasmids were transfected to HEK293T cells and western blots against EGFP and different tags are shown. **(C)** siRNA against PIAS1, PIAS2 and PIAS3 was transfected to HEK293T cells and western blots against Flag and PIAS proteins (PIAS1, PIAS2 and PIAS3) are shown. **(D)** Flag-Hes-1WT and EGFP-PIAS1WT plasmids were co-transfected to HEK293T cells and confocol image showing the sub-cellular localization of Hes-1 (red) and PIAS1 (green). The nuclei were visualized with DAPI staining (blue). Scale bar equals 10 μm. **(E)** Flag-Hes-1WT and EGFP-PIAS1WT plasmids were transfected to HEK293T cells followed by immunoprecipitation and western blot showing Hes-1 association with PIAS1 (left panel) and *vice versa* (right panel). **(F)** Various combinations of Flag-Hes-1, Myc-SUMO-1, EGFP-PIAS1WT or EGFP-PIAS1W372A plasmid were co-transfected to HEK293T cells and the cell lysate was subject to western blot against various tags. The sumoylated Hes-1 bands are shown in the bracket. Each experiment was performed twice.

Next, we examined whether Hes-1 SUMOylation is altered in response to external stimuli and here, H_2_O_2_ was used as a stimulus. The reason to use H_2_O_2_ is because that H_2_O_2_ causes oxidative stress in many cell lines studied and that H_2_O_2_ induces global protein SUMOylation [22]. Flag-Hes-1WT plasmid was transfected to HEK293T cells with the addition of different concentrations of H_2_O_2_. The cell lysate was immunoprecipitated with anti-Flag antibody and immunoblotted with anti-Hes-1 and anti-SUMO-1 antibodies. Results revealed that H_2_O_2_ (ranging from 0.01 mM to 0.5 mM) produced an apparent and dose-dependent increase in Hes-1 SUMOylation (Figure 3A). Further time-course study revealed that H_2_O_2_ (0.2 mM) produced a time-dependent increase in Hes-1 SUMOylation up to 4 h after H_2_O_2_ treatment (Figure 3B). We then examined whether SUMO-modification of endogenous Hes-1 could be observed under H_2_O_2_ treatment. HEK293T cells were transfected with Myc-SUMO-1 plasmid with or without H_2_O_2_ co-treatment. The cell lysates were immunoprecipitated with anti-Myc antibody and immunoblotted with anti-Hes-1 and anti-SUMO-1 antibody. Results revealed that endogenous Hes-1 SUMOylation was consistently observed upon Myc-SUMO-1 transfection, but H_2_O_2_ further enhanced the SUMOylation of Hes-1 (Figure 3C). We next examined whether H_2_O_2_ increases the association between PIAS1 and Hes-1, which would result in enhanced Hes-1 SUMOylation, and whether this is PIAS1 phosphorylation-dependent. H_2_O_2_ was added to HEK293T cells for different time periods and results indicated that H_2_O_2_ treatment increased the phosphorylation level of PIAS1 at Ser90, and this effect was most apparent 0.5 h later; but H_2_O_2_ did not alter the expression level of PIAS1 (Figure 3D, upper panel). Next, Flag-Hes-1WT plasmid was co-transfected with EGFP-PIAS1WT plasmid or the phosphorylation mutant plasmid EGFP-PIAS1S90A to HEK293T cells together with the addition of H_2_O_2_, and the interaction between PIAS1 and Hes-1 was examined 4 h after H_2_O_2_ treatment. Results revealed that H_2_O_2_ apparently increased the association between PIAS1 and Hes-1 (lane 2 vs. lane 5), but this effect was diminished when EGFP-PIAS1S90A, instead of EGFP-PIAS1WT, was transfected (lane 5 vs. lane 6) (Figure 3D, lower panel). The role of PIAS1 phosphorylation was further confirmed by the result that treatment of CIP, a phosphatase, reduced the association between PIAS1 and Hes-1 that was induced by H_2_O_2_ treatment (lane 4 vs. lane 6) (Figure 3E). In addition, immunoblot against Flag (longer exposure) showed the absence of the slowly migrating bands just above 43 kDa in the presence of CIP.

### Identification of the major SUMO acceptors on Hes-1

**Figure 4:**
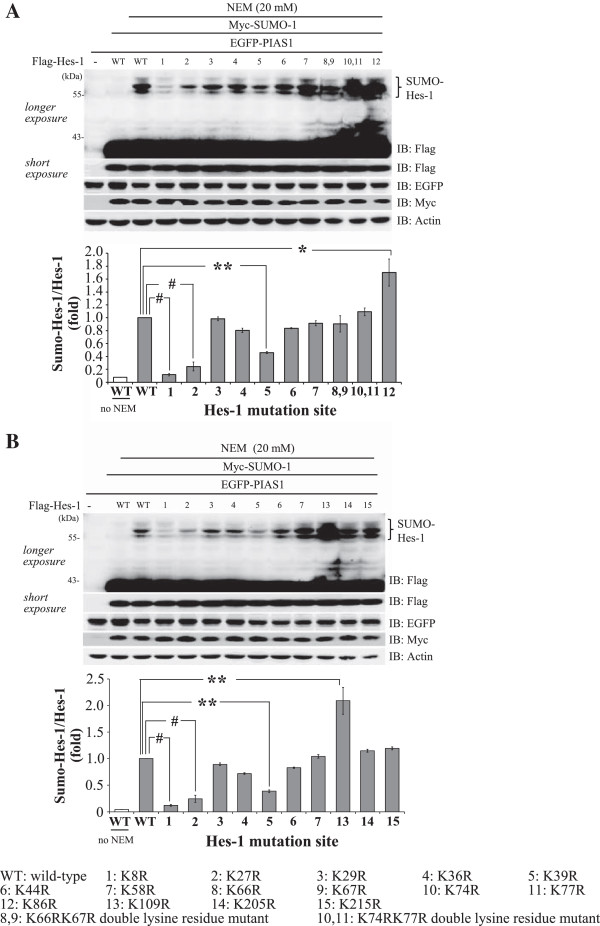
**Identification of candidate SUMO sites on Hes-1.** Flag-Hes-1WT plasmid or individual Flag-Hes-1 lysine residue mutant plasmid was co-transfected with Myc-SUMO-1 and EGFP-PIAS1 to HK293T cells and the cell lysate was subject to western blot against various tags. **(A)** Gel pattern showing Hes-1 SUMOylation upon transfection of Hes-1 lysine residue mutant from Lys8 (K8R) to Lys86 (K86R). The quantification result is shown in the lower panel. **(B)** Gel pattern showing Hes-1 SUMOylation upon transfection of Hes-1 lysine residue mutant from Lys109 (K109R) to Lys215 (K215R). Lysine residue mutants from Lys8 to Lys58 were repeated for the purpose of comparison. The quantification result is shown in the lower panel. Results were obtained from two independent experiments. The sumoylated Hes-1 bands are shown in the bracket. Data are expressed as mean ± SEM (standard error of mean). *p < 0.05, **p < 0.01 and # *p* < 0.001.

**Figure 5:**
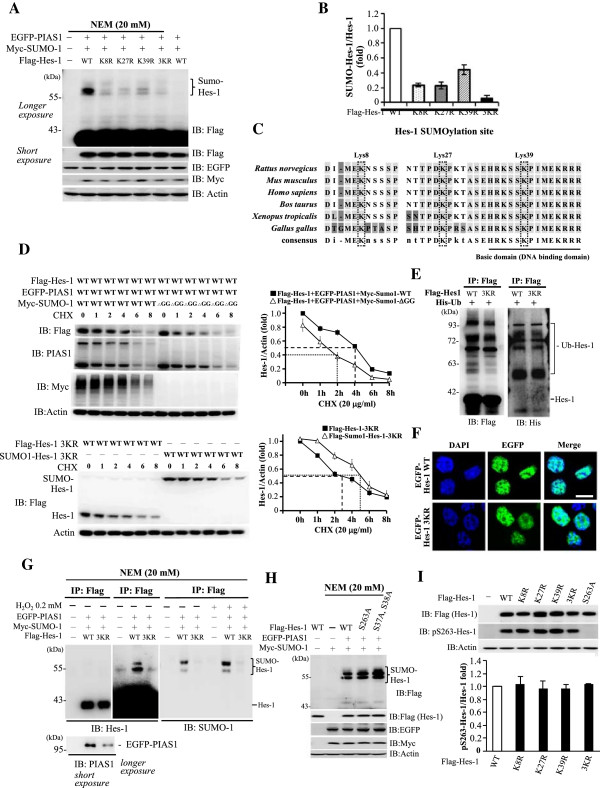
**Hes-1 SUMOylation stabilizes Hes-1, and Hes-1 SUMOylation and Hes-1 phosphorylation are independent each other. (A)** EGFP-PIAS1WT and Myc-SUMO-1 were co-transfected with Flag-Hes-1WT or Flag-Hes-1 sumo-mutant for immunoblot analysis against various tags. The quantitative result was shown in **(B)**. **(C)** Sequence alignment showing amino acid sequences around the three SUMOylation sites on Hes-1 in different species of animals by using the ClustalW web-based tool. **(D)** Flag-Hes-1WT and EGFP-PIAS1WT were co-transfected with Myc-SUMO-1 or Myc-SUMO-1ΔGG to cells and cycloheximide was added to the cell for different time periods (upper panel). In addition, Flag-Hes-1 3KR or Flag-SUMO-1-Hes-1 3KR was transfected to HEK293T cells and cycloheximide was added to the cell for different time periods (lower panel). The cell lysate was subject to immunoblot using antibody as indicated. A representative gel pattern and the quantitative result are shown. **(E)** Flag-Hes-1WT or Flag-Hes-1 3KR was co-transfected with His-ubiquitin to HEK293T cells. The cell lysate was immunoprecipitated with anti-Flag antibody and immunoblotted with antibodies against Flag or His. **(F)** Confocol images show the localization of EGFP-Hes-1WT and EGFP-Hes-1 3KR and co-localization with DAPI. Scale bar equals 10 μm. **(G)** EGFP-PIAS1WT and Myc-SUMO-1 were co-transfected with Flag-Hes1WT or Flag-Hes-1 3KR to HEK293T cells with or without H_2_O_2_ treatment. The cell lysate was immunoprecipitated with anti-Flag antibody followed by immunoblot against Hes-1 (left and middle panels), SUMO-1 (right panel), and PIAS1 (left-lower panel). **(H)** EGFP-PIAS1WT and Myc-SUMO-1 were transfected with wild-type or phosphorylation site mutant plasmids into HEK293T cell, and the cell lysate was subject to immunoblot against various tags. **(I)** Flag-Hes-1WT, Flag-Hes-1-S263A or different Flag-Hes-1 sumo-mutant was transfected to HEK293T cells for immunoblot against Flag or pSer263-Hes-1. The quantified results are shown (lower panel). The sumoylated or Ubiquitinated Hes-1 bands are shown in the bracket. Each experiment was performed twice. CHX: cycloheximide, Ub: ubiquitin.

There are 15 lysine residues on the Hes-1 protein; however, no consensus SUMO-substrate motif (ψ-K-X-E, where ψ stands for a hydrophobic amino acid) was found on Hes-1, therefore, we have constructed individual lysine residue mutant for identification of the target SUMOylation site(s) on Hes-1 in the cell. Results revealed that PIAS1 consistently sumoylated Hes-1, but the intensity of Hes-1 SUMOylation was significantly decreased when Flag-Hes-1K8R, Flag-Hes-1K27R or Flag-Hes-1K39R was transfected (*p* < 0.001, *p* < 0.001 and *p* < 0.01, respectively) (Figure 4A and B). On the other hand, transfection of Flag-Hes-1K86R and Flag-Hes-1K109R both increased the intensity of Hes-1 SUMOylation (*p* < 0.05 and *p* < 0.01, respectively) (Figure 4A and B). Because Hes-1 could be ubiquinated at both K86 and K109 [23], mutation of Hes-1 at these two residues would decrease the ubiquitylation of Hes-1 that may result in the stabilization of Hes-1 and consequently, the elevation of Hes-1 SUMOylation. Based on the above results, we then generated the Hes-1 triple sumo-mutant construct targeted to K8, K27 and K39 residues (Hes-1 3KR) and further examined the effects of Hes-1K8R, Hes-1K27R, Hes-1K39R and Hes-1 3KR on Hes-1 SUMOylation. Results revealed that transfection of each Hes-1 sumo-mutant significantly decreased the level of Hes-1 SUMOylation (all *p* < 0.01), but this effect was almost completely abolished when Flag-Hes-1 3KR was transfected (*p* < 0.001) (Figure 5A and B). Alignment of the amino acid sequence of Hes-1 indicated that Lys8, Lys27 and Lys39 of Hes-1 are highly conserved in different species of the vertebrate (Figure 5C).

### Hes-1 SUMOylation stabilizes the Hes-1 protein

In this series of experiments we examined whether Hes-1 SUMOylation affects the stability of Hes-1. Flag-Hes-1WT plasmid was co-transfected with EGFP-PIAS1WT plasmid and Myc-SUMO-1WT plasmid or Myc-SUMO-1ΔGG plasmid to HEK293T cells and the cells were treated with cycloheximide (50 μg/ml) for different time periods. Cell lysates were subject to SDS-PAGE followed by immunoblot with anti-Flag antibody (Figure 5D, upper-left panel). Results revealed that sumoylated Hes-1 degraded slower with a half-life of 4 h approximately, whereas the un-sumoylated Hes-1 showed a half-life for about 2 h (Figure 5D, upper-right panel). Next, we have transfected Flag-Hes-1 3KR or Flag-SUMO-1-fusioned Hes-1 3KR plasmid to HEK293T cells and the cells were treated with cycloheximide (50 μg/ml) for different time periods. Cell lysates were similarly subject to SDS-PAGE and immunoblotted with anti-Flag antibody (Figure 5D, lower left panel). Results revealed that SUMO-1-fusioned Hes-1 3KR protein showed a slower degradation rate with a half-life for about 5 h, whereas the Hes-1 3KR protein showed a half-life for about 2.8 h (Figure 5D, lower-right panel). Because lysine residues are subject to both SUMOylation and ubiquitination modifications, we further examined whether Hes-1WT and Hes-1 3KR proteins may have a different ubiquination level. Flag-Hes-1WT or Flag-Hes-1 3KR plasmid was co-transfected with His-ubiquitin plasmid to HEK293T cells and the cell lysates were immunoprecipitated with anti-Flag antibody followed by immunoblotting with anti-Flag and anti-His antibody. Results revealed that the Hes-1WT and Hes-1 3KR proteins showed a similar ubiquitination level (Figure 5E, right panel). In addition, we have also examined whether Hes-1 3KR is correctly localized in the cell. EGFP-tagged Hes-1WT and Hes-1 3KR plasmids were transfected to HEK293T cells and the cells were stained with DAPI (blue). Results revealed that Hes-1 3KR showed the same localization as Hes-1 WT does and the merged images indicated that they both are localized in the nucleus (Figure 5F).

PIAS1 SUMOylation of Hes-1 at these three lysine residues was further examined here. Flag-Hes-1WT or Flag-Hes-1 3KR plasmid was co-transfected with EGFP-PIAS1 and Myc-SUMO-1 to HEK293T cells. Cells were lysed with RIPA buffer containing 20 mM NEM and subject to immunoprecipitation followed by immunoblotting. Results revealed that transfection of either Flag-Hes-1WT or Flag-Hes-1 3KR yielded approximately equal amount of the Hes-1 protein when anti-Hes-1 antibody was used (Figure 5G, left panel), but the association between Hes-1 and PIAS1 was reduced when Flag-Hes-1 3KR was transfected (Figure 5G, lower panel). Furthermore, co-transfection of EGFP-PIAS1, Myc-SUMO-1 with Flag-Hes-1WT showed apparent bands for sumoylated Hes-1 when immunoblotted with anti-Hes-1 antibody and anti-SUMO-1 antibody, but Hes-1 SUMOylation was abolished when Flag-Hes-1 3KR was transfected (Figure 5G, middle and right panels). Hes-1 SUMOylation was further enhanced upon H_2_O_2_ treatment (lane 2 vs. lane 5, Figure 5G, right panel), but this effect was completely abolished when Flag-Hes-1 3KR was transfected (lane 5 vs. lane 6, Figure 5G, right panel).

### Hes-1 SUMOylation is Hes-1 phosphorylation-independent and *vice versa*

Interplay between protein SUMOylation and protein phosphorylation has been suggested [24,25]. Here, we examined whether Hes-1 SUMOylation is Hes-1 phosphorylation-dependent. The Hes-1WT and Hes-1 phosphorylation mutant plasmids at Ser37, Ser38 and Ser263 were transfected to HEK293T cells with the co-transfection of EGFP-PIAS1 and Myc-SUMO-1. The cell lysates were subject to immunoprecipitation and immunoblotting. Results revealed that PIAS1 consistently sumoylated Hes-1, but Hes-1 SUMOylation was not affected when Hes-1S263A and Hes-1S37AS38A were transfected (Figure 5H). We further examined whether SUMOylation of Hes-1 affects the phosphorylation of Hes-1. Flag-Hes-1WT and different Flag-Hes-1 sumo-mutant plasmids were transfected to HEK293T cells and the cell lysates were subject to western blot. Results revealed that transfection of any Hes-1 sumo-mutant did not affect the phosphorylation level of Hes-1 at Ser263. Transfection of Hes-1S263A was used as a negative control (Figure 5I).

### Hes-1 binds to the GADD45α promoter and blockade of Hes-1 SUMOylation reduces Hes-1 binding to GADD45α and decreases Hes-1 suppression of GADD45α expression

**Figure 6:**
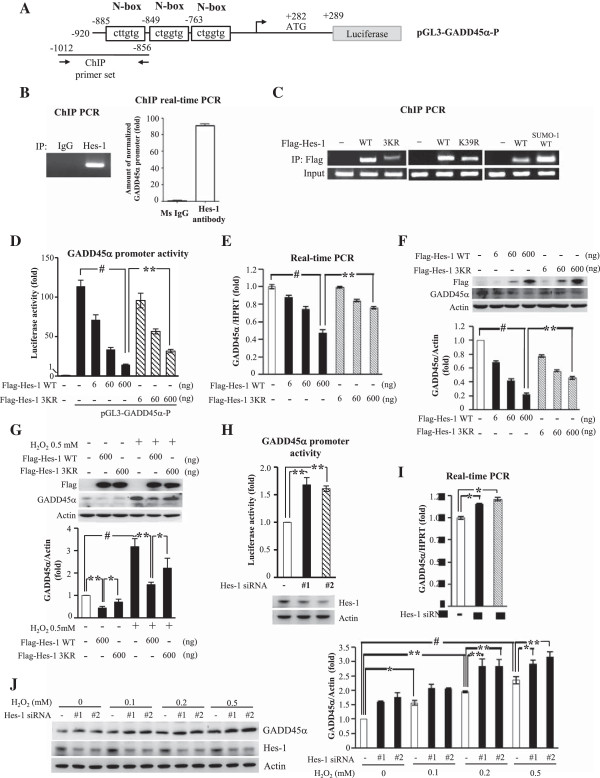
**Hes-1 binds to the GADD45α promoter and blockade of Hes-1 SUMOylation reduces Hes-1 binding to GADD45α and decreases Hes-1 suppression of GADD45αexpression. (A)** pGL3-GADD45α-P: cloned GADD45α promoter. N-boxes and primers used for Hes-1 ChIP assay were shown. **(B)** ChIP PCR was performed using Hes-1 antibody (or mouse IgG) and the primer for GADD45α promoter. The PCR product (left panel) and the result of ChIP real-time PCR (right panel) were shown. **(C)** Flag-Hes-1WT, Flag-Hes-1 3KR or Flag-SUMO-1-fusioned Hes-1WT was transfected to cells for ChIP PCR with anti-Flag antibody and primers for GADD45α promoter. Input: PCR product from input lysate. **(D)** Different doses of Flag-Hes-1WT or Flag-Hes-1 3KR was co-transfected with pGL3-GADD45α-P to cells for promoter activity assay. Different doses of Flag-Hes-1WT or Flag-Hes-1 3KR was transfected to cells and GADD45α mRNA **(E)** and protein **(F)** expression were analyzed by real-time PCR and immunoblot respectively, and the quantified results were shown. **(G)** Flag-Hes-1WT or Flag-Hes-1 3KR was transfected to cells with or without H_2_O_2_ treatment followed by immunoblot against GADD45α and Flag. The quantified result is shown below. **(H)** Two sets of Hes-1 siRNA and 0.4 μg of pGL3-GADD45α-P were co-transfected to cells for promoter activity assay. Two sets of Hes-1 siRNA were transfected to cells with or without the addition of H_2_O_2_ for GADD45α mRNA **(I)** and protein **(J)** expression analysis by real-time PCR and immunoblot against GADD45α and Flag respectively. Data for luciferase activity and western blot are expressed as that in Figure 4. Values for mRNA measure are averaged from two independent experiments with mean value for the control group normalized to 1.0. Other values are normalized to the control value. Each experiment was performed twice. * *p* < 0.05, ** *p* < 0.01 and # *p* < 0.001.

SUMOylation of transcription factors has been shown to affect their DNA-binding activity [26]. Hes-1 SUMOylation has not been reported previously, but Hes-1 was found to mediate cell survival upon amyloid-beta insult [27]. However, the underlying mechanism of the protective effect of Hes-1 is not fully understood. Here we aimed to examine whether Hes-1 SUMOylation may affect cell survival through alteration of Hes-1 binding to DNA promoter and downstream gene expression. A variety of genes are known to be responsive to Hes-1 [28]. There are also many known stress sensors in the cell. In considering both factors we have carried out a preliminary study examining the effects of knockdown of Hes-1 on the expression of E2F1 and *G*rowth *A*rrest and *D*NA *D*amage-inducible 45α (GADD45α). It turned out that Hes-1 had a more apparent effect in suppressing GADD45α expression. In addition, PIAS1 was found to negatively regulate the expression of GADD45α [29]. Based on these results, we have therefore chosen GADD45α, an important stress sensor in the cell [30], for the present study. GADD45α transcript can be rapidly induced by many types of DNA-damaging agents including UV-irradiation [31], alkylation agent [32], and oxidizing agents such as H_2_O_2_[33-35]. Accumulative evidence indicates that GADD45α is involved in a wide range of biological processes, including maintenance of genomic stability, differentiation, senescence, and apoptosis [36]. Bioinformatic analysis indicated that there are three N-boxes (CACNAG), which is specific for Hes-1 binding, within 1 kb from the transcription start sites of human GADD45α promoters (nt -885 ~ nt -880, nt -849 ~ nt -844 and nt -763 ~ nt -758) (Figure 6A). To study whether Hes-1 actually binds to the GADD45α promoter, ChIP assay was performed. Results showed that Hes-1 apparently binds to the GADD45α promoter (Figure 6B). The PCR product (156 bp) from ChIP assay was also observed by ChIP-PCR and DNA electrophoresis (Figure 6B, left panel).

Among the three SUMOylation residues identified on Hes-1, Lys39 is located in the basic domain of Hes-1, which confers Hes-1 DNA-binding activity during transcriptional repression [37]. This suggests that SUMOylation of Hes-1 may be associated with the DNA binding activity of Hes-1. To test this hypothesis, the DNA- binding activity of Hes-1WT and Hes-1 3KR was examined by ChIP-PCR. Results revealed that Hes-1WT consistently binds to the GADD45α promoter, but Hes-1 3KR apparently decreased the binding activity to the GADD45α promoter (Figure 6C, left panel). We next examined the effect of mutation of Lys39 alone on Hes-1 DNA- binding. Result revealed that mutation of Lys39 alone decreased Hes-1 binding to GADD45α promoter for approximately 55%, but it is not sufficient to block Hes-1 DNA-binding (Figure 6C, middle panel). This latter result suggests that Lys8 and Lys27, the target sumo sites on Hes-1, also play an important role in Hes-1 DNA binding. To further address this issue, we have examined the DNA binding activity of Hes-1WT and SUMO-1-fusioned Hes-1. Result from ChIP-PCR revealed that SUMO-1-fusioned Hes-1 showed approximately three-fold increase in DNA binding to GADD45α promoter than Hes-1WT did (Figure 6C, right panel). Next, we examined whether blockade of Hes-1 SUMOylation affects the promoter activity of GADD45α. Human GADD45α promoter (nt -920 to nt +289) was cloned from the genomic library of HEK293T cells and co-transfected with different doses of the Flag-Hes-1WT or Flag-Hes-1 3KR plasmid to HEK293T cells for luciferase reporter assay. Results indicated that Flag-Hes-1WT dose-dependently suppressed GADD45α promoter activity (*p* < 0.05, *p* < 0.01 and *p* < 0.001), but this effect was diminished by Flag-Hes-1 3KR also in a dose-dependent manner (*p* < 0.05 or *p* < 0.01) (Figure 6D). Furthermore, results from quantitative PCR indicated that transfection of Flag-Hes-1WT plasmid decreased GADD45α mRNA level dose-dependently (*p* < 0.05, *p* < 0.01 and *p* < 0.001), but this effect was partially reversed by Flag-Hes-1 3KR transfection (*p* < 0.01 for 600 ng dose) (Figure 6E). The same results were found with GADD45α protein expression (*p* < 0.001 and *p* < 0.01) (Figure 6F). Plasmid transfection and expression was confirmed by western blot against Flag (Figure 6F). Because GADD45α is a stress sensor, next we examined the effect of Hes-1 SUMOylation on GADD45α protein expression under the challenge of H_2_O_2_. Results revealed that H_2_O_2_ dramatically increased the expression of GADD45α (*p* < 0.001). This effect was decreased by Flag-Hes-1WT transfection (*p* < 0.01), but Flag-Hes-1 3KR was less able to produce the same effect (*p* < 0.05 compared with the Flag-Hes-1WT group) (Figure 6G). Plasmid transfection and expression was confirmed by western blot against Flag (Figure 6G).

Because Hes-1 suppressed GADD45α promoter activity and expression, we expect that knockdown of Hes-1 expression should increase GADD45α promoter activity and expression. This issue was examined here. Two different sets of Hes-1 siRNA were transfected to HEK293T cells, respectively. Results revealed that both Hes-1 siRNA transfections increased GADD45α promoter activity (both *p* < 0.01) (Figure 6H) and endogenous GADD45α mRNA level (both *p* < 0.05) (Figure 6I). We further examined whether knockdown of Hes-1 also increases GADD45α protein expression under H_2_O_2_ challenge. Both sets of Hes-1 siRNA were transfected to HEK293T cells, respectively, with the addition of different concentrations of H_2_O_2_. Results revealed that H_2_O_2_ produced a dose-dependent increase in GADD45α protein expression (*p* < 0.05, *p* < 0.01 or *p* < 0.001), and both Hes-1 siRNA transfections increased GADD45α protein expression under each dose of H_2_O_2_ examined (*p* < 0.05 or *p* < 0.01) (Figure 6J). A representative gel pattern from western blot is shown (Figure 6J, left panel).

### PIAS1 enhances the suppressing effect of Hes-1 on GADD45α expression that is blocked by Hes-1 3KR

**Figure 7:**
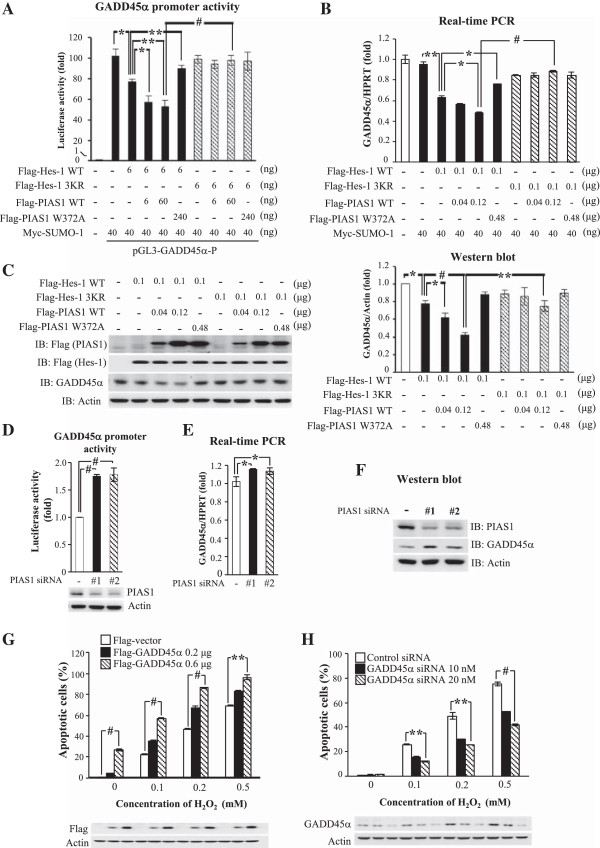
**PIAS1 enhances the suppressing effect of Hes-1 on GADD45α expression that is blocked by Hes-1 3KR.** Different doses of Flag-PIAS1WT or Flag-PIAS1W372A plasmid was co-transfected with Myc-SUMO-1 and various doses of Flag-Hes-1WT or Flag-Hes-1 3KR plasmid together with 0.4 μg of GADD45α promoter construct to HEK293T cells for determination of **(A)** GADD45α promoter activity and **(B)** GADD45α mRNA level by real-time PCR. **(C)** Different doses of Flag-PIAS1WT or Flag-PIAS1W372A plasmid was co-transfected with various doses of Flag-Hes-1WT or Flag-Hes-1 3KR plasmid to HEK293T cells and western blot against GADD45α and Flag was performed. The quantified result is shown on the right panel. **(D)** Two sets of PIAS1 siRNA and 0.4 μg of GADD45α promoter construct were transfected to HEK293T cells and GADD45α promoter activity was determined 48 h later. Two sets of PIAS1 siRNA were transfected to HEK293T cells and **(E)** GADD45α mRNA level and **(F)** GADD45α protein level were determined 48 h later. The effectiveness of PIAS1 siRNA transfection is confirmed by western blot against PIAS1. **(G)** Two doses of Flag-GADD45α plasmid was transfected to cells with the addition of different concentrations of H_2_O_2_ and cell apoptosis was determined by TUNEL assay. Western blot against the Flag-tag confirms plasmid transfection and expression. **(H)** Two concentrations of GADD45α siRNA was transfected to HEK293T cells with the addition of different concentrations of H_2_O_2_ and cell apoptosis was determined by TUNEL assay. Western blot against GADD45α confirms the effectiveness of GADD45α siRNA transfection. Data are expressed as that in Figure 6. Each experiment was performed twice. *p < 0.05, **p < 0.01 and # *p* < 0.001.

The above results showed that blockade of Hes-1 SUMOylation decreased the transcriptional suppression activity of Hes-1 on GADD45α expression. Here we further examined the role of Hes-1 SUMOylation in regulation of GADD45α promoter activity and expression by overexpression of SUMO-1, PIAS1WT or PIAS1W372A, Hes-1WT or Hes-1 3KR to HEK293T cells. Results revealed that transfection of Flag-Hes-1WT plasmid consistently suppressed GADD45α promoter activity (*p* < 0.05) (Figure 7A, lane 3 vs. lane 2), and co-transfection of Flag-PIAS1 further enhanced the suppressing effect of Hes-1 (*p* < 0.05 and *p* < 0.01 for 6 ng and 60 ng of Flag-PIAS1) (Figure 7A, lane 4, 5 vs. lane 3), but co-transfection of Flag-PIAS1W372A antagonized the effect of Hes-1 on GADD45α promoter activity (*p* < 0.01) (Figure 7A, lane 6 vs. lane 3). Further, the enhancing effect of Flag-PIAS1 was blocked by co-transfection of Flag-Hes-1 3KR (*p* < 0.05 or *p* < 0.001) (Figure 7A, lane 7-9 vs. lane 3-5). The same plasmid transfection yielded similar results on GADD45α mRNA expression (*p* < 0.05, *p* < 0.01 or *p* < 0.001) (Figure 7B) and protein expression (*p* < 0.05, *p* < 0.01 or *p* < 0.001) (Figure 7C). Plasmid transfection and expression was confirmed by western blot against Flag (Figure 7C).

Results from Figure 6D and E showed that Hes-1 negatively regulates GADD45α promoter activity and mRNA expression, here we further examined the role of PIAS1 in regulation of GADD45α expression. Two sets of PIAS1 siRNA were transfected to HEK293T cells to study this issue. Results revealed that both sets of PIAS1 siRNA transfection increased GADD45α promoter activity (both *p* < 0.001) (Figure 7D). Both PIAS1 siRNA transfetions also decreased PIAS1 expression (Figure 7D, lower panel). Similarly, both sets of PIAS1 siRNA transfection increased GADD45α mRNA level (both *p* < 0.05) (Figure 7E) and GADD45α protein level (Figure 7F). The effectiveness of both PIAS1 siRNA transfections was confirmed by a decreased level of PIAS1 expression from western blot (Figure 7F).

### Effects of GADD45α on H_2_O_2_-induced cell apoptosis

It is shown that GADD45α is involved in cell apoptosis [38,39] or anti-apoptosis [40], and this effect is dependent upon the stimulus and cell type studied. Here we examined whether GADD45α produces toxicity to HEK293T cells and whether GADD45α potentiates the effect of H_2_O_2_ on cell apoptosis. Results from TUNEL (*t*erminal deoxynucleotidyl transfease d*U*TP *n*ick-*e*nd *l*abeling) assay revealed that transfection of Flag-GADD45α caused cell apoptosis in a dose-dependent manner (*p* < 0.01 for 0.6 μg), and it further potentiated H_2_O_2_-induced apoptosis (*p* < 0.001 or *p* < 0.01) (Figure 7G). Furthermore, the toxicity of H_2_O_2_ was attenuated by transfection of GADD45α siRNA in a dose-dependent manner (*p* < 0.01 or *p* < 0.001) (Figure 7H). Plasmid and siRNA transfection and protein expression were confirmed by western blot using anti-Flag antibody (Figure 7G, lower panel) and anti-GADD45α antibody (Figure 7H, lower panel).

### Hes-1 and PIAS1 protect against H_2_O_2_-induced apoptosis through Hes-1 SUMOylation

**Figure 8:**
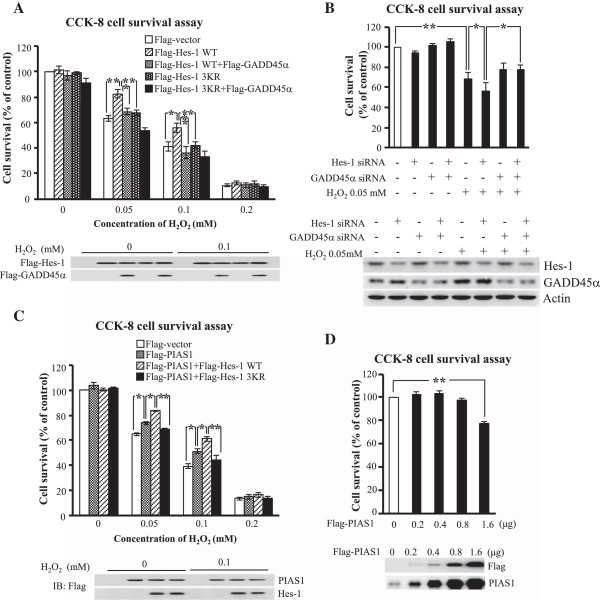
**Hes-1 and PIAS1 both protect against H**_**2**_**O**_**2**_**-induced apoptosis through SUMOyaltion of Hes-1, and high dose of PIAS1 decreases cell survival. (A)** Flag-Hes-1WT or Flag-Hes-1 3KR plasmid, alone or in combination with Flag-GADD45α, was transfected to HEK293T cells together with different concentrations of H_2_O_2_ for examination of cell survival by using CCK-8 activity assay. Western blot against the Flag-tag confirms the transfection and expression of Hes-1 plasmid and GADD45α plasmid, and a representative gel pattern under 0.1 mM H_2_O_2_ treatment is shown (lower panel). **(B)** Hes-1 siRNA (20 nM), alone or together with GADD45α siRNA, was transfected to HEK293T cells with or without the addition of H_2_O_2_ (0.05 mM) and cell survival was determined by CCK-8 activity assay. The effectiveness of Hes-1 siRNA and GADD45α siRNA transfections was confirmed by western blot against Hes-1 and GADD45α. **(C)** Flag-PIAS1WT plasmid, alone or in combination with Flag-Hes-1WT or Flag-Hes-1 3KR plasmid was transfected to HEK293T cells together with different concentrations of H_2_O_2_ for examination of cell survival by using CCK-8 activity assay. Western blot against the Flag-tag confirms the transfection and expression of PIAS1 plasmid and Hes-1 plasmid, and a representative gel pattern under 0.1 mM H_2_O_2_ treatment is shown (lower panel). **(D)** Different doses of Flag-PIAS1WT plasmid were transfected to HEK293T cells and cell viability was determined by CCK-8 activity assay. Flag-PIAS1WT transfection and expression was confirmed by western blot against PIAS1 and Flag. Data are expressed as mean ± SEM from two independent experiments. *p < 0.05 and **p < 0.01.

The above results showed that Hes-1 suppressed GADD45α promoter activity and GADD45α expression and these effects were alleviated by transfection of the Hes-1 sumo-mutant Hes-1 3KR. In addition, GADD45α yielded cell apoptosis in HEK293T cells. In this experiment, we examined whether Hes-1 exerts a protective effect against H_2_O_2_-induced apoptosis and whether this effect is also blocked by Hes-1 3KR. Because previous results revealed that H_2_O_2_ ranging from 0.01 mM to 0.5 mM produced consistent and dose-dependent increase in Hes-1 SUMOylation (Figure 2A), we have adopted concentrations of H_2_O_2_ ranging from 0.05 mM to 0.2 mM for the present experiment. Various Flag-tagged Hes-1 plasmids and GADD45α plasmid were transfected to HEK293T cells and their effects on cell survival were determined by CCK-8 assay upon H_2_O_2_ challenge. Results revealed that H_2_O_2_ consistently decreased cell survival in a dose-dependent manner (*p* < 0.001). Overexpression of Hes-1 protected against this effect of H_2_O_2_ (for 0.05 mM and 0.1 mM of H_2_O_2_) (*p* < 0.05 or *p* < 0.01). But the protective effect of Hes-1 was diminished by co-transfection of GADD45α (for 0.05 mM and 0.1 mM of H_2_O_2_) (*p* < 0.05 or *p* < 0.01). Furthermore, the protective effect of Hes-1 was no longer observed when Flag-Hes-1 3KR, instead of Flag-Hes-1WT, was transfected (for 0.05 mM and 0.1 mM of H_2_O_2_) (*p* < 0.01) (Figure 8A). When the concentration of H_2_O_2_ is too high (0.2 mM), neither the protective effect of Hes-1 nor the pro-apoptotic effect of GADD45α and Hes-1 3KR was observed (*p* > 0.05). Plasmid transfection and expression was confirmed by western blot against Flag and a representative gel pattern for control and 0.1 mM H_2_O_2_ is shown (Figure 8A, lower panel). On the other hand, knockdown of Hes-1 expression by Hes-1 siRNA transfection at a concentration that did not produce a significant effect alone, potentiated H_2_O_2_-induced decrease in cell survival (*p* < 0.05) (Figure 8B). But knockdown of GADD45α expression suppressed the decreased cell survival seen with Hes-1 siRNA transfection (*p* < 0.05) (Figure 8B). The effectiveness of Hes-1 siRNA and GADD45α siRNA transfection was confirmed by decreased Hes-1 and GADD45α expression, respectively, from western blot (Figure 8B, lower panel).

### PIAS1 protects against H_2_O_2_-induced apoptosis through SUMOylation of Hes-1

The above results showed that Hes-1 protects against H_2_O_2_-induced apoptosis and this effect was prevented by Hes-1 3KR. Here we examined whether enhanced SUMOylation of Hes-1 facilitates the protective effect of Hes-1. The Flag-PIAS1 plasmid was transfected alone or co-transfected with Flag-Hes-1WT or Flag-Hes-1 3KR plasmid to HEK293T cells and their effects on cell survival were determined by CCK-8 assay upon H_2_O_2_ insult. Results revealed that H_2_O_2_ consistently decreased cell survival in a dose-dependent manner (*p* < 0.001). Overexpression of PIAS1 protected against this effect of H_2_O_2_ (*p* < 0.05). The protective effect of PIAS1 was further enhanced by Flag-Hes-1WT co-transfection (*p* < 0.05), but this enhancing effect of Hes-1 was blocked by Flag-Hes-1 3KR co-transfection (*p* < 0.01) (Figure 8C). Similarly, the protective effect of PIAS1 and Hes-1 and the blockade effect of Hes-1 3KR were not observed when the concentration of H_2_O_2_ is too high (0.2 mM) (*p* > 0.05). Plasmid transfection and expression was confirmed by western blot against Flag and a representative gel pattern for control and 0.1 mM H_2_O_2_ is shown (Figure 8C, lower panel). Furthermore, we examined the effect of PIAS1 overexpression on cell survival. Different amount of Flag-PIAS1 plasmid was transfected to HEK293T cells and cell survival was determined by CCK-8 assay 48 h later. Results revealed that transfection of Flag-PIAS1 from 0.2 μg to 0.8 μg did not produce an effect on cell survival (*p* > 0.05). However, transfection of Flag-PIAS1 at 1.6 μg apparently decreased cell survival (*p* < 0.01) (Figure 8D). PIAS1 plasmid transfection and expression was confirmed by immunoblot against Flag and PIAS1 (Figure 8D, lower panel).

## Discussion

In this study, we have identified the transcriptional repressor Hes-1 as a novel SUMO substrate. In the cell, endogenous Hes-1 SUMOylation was not readily observed due to low Hes-1 antibody efficiency and very few amount of IP product obtained. Thus, overexpression of Myc-SUMO-1 (but not overexpression of Hes-1) was adopted which allows the detection of Hes-1 SUMOylation (Figure 1D), suggesting that endogenous Hes-1 SUMOylation does take place in the cell. However, it is known that the SUMO molecule is attached to most substrates at the lysine residue (K) of the ψ-K-X-E consensus motif, which is directly bound by the E2 ligase UBC9. This direct interaction explains why E1 and E2 only are sufficient to sumoylate many substrates at the correct lysine residue in the absence of any E3 [6]. Actually, SUMO attach at non-consensus sites has also been reported and it is suggested that the E3 ligase activity is particularly important for SUMOylation at atypical consensus motif [6,41]. Therefore, the association of E3 ligase and Hes-1 was further examined in our study. In support of this hypothesis, we have found that PIAS1 greatly enhanced the SUMOylation of Hes-1, and this effect was abolished by both PIAS1 siRNA and PIAS1W372A transfections. Furthermore, PIAS2 and PIAS3 also apparently increased the SUMOylation of Hes-1, but RanBP2 and Pc2 did not affect the SUMOylaiton of Hes-1. These results together reveal the important role of the PIAS family E3 ligase in Hes-1 SUMOylation in the cell.

There are seven members of the Hes protein in the Hes protein family (Hes-1 to Hes-7) [28]. Among these Hes proteins, Hes-1 and Hes-5 are important Notch effectors. Once activated by Delta, the NICD is cleaved by γ-secretase and leads to the induction of Hes-1 and Hes-5 expression [42,43]. In the absence of Hes-1 and Hes-5, NICD is unable to inhibit neurogenesis [1]. Studies using knockout mice have shown that Hes-1 and Hes-5 operate in a common signaling pathway and they functionally compensate each other [44,45]. However, in the present study we have found that Hes-1 could be SUMO modified by PIAS1, but Hes-5 could not. In another study, we have found that Hes-1 strongly regulates GluR1 expression in cultured cortical neurons but Hes-5 only moderately does so [3]. These results together suggest that although both Hes-1 and Hes-5 are Notch effectors, their post-translational modifications could be different and it is conceivable that they also participate in different cellular functions.

In the present study, we have identified Lys8, Lys27 and Lys39 as the major SUMO sites on Hes-1 and mutation at these residues significantly decreased the DNA binding activity of Hes-1 to GADD45α promoter. Among these three lysine residues, Lys39 is located in the basic domain of GADD45α and Hes-1 can directly bind to DNA through its basic domain. Sequence alignment indicated that Lys39 of Hes-1 is highly conserved in different species of vertebrates. Facilitation of DNA binding upon protein SUMOylation has also been demonstrated for other transcription factors. For example, SUMOylation of heat shock transcription factor 2 (HSF2) at Lys82, which is also located in the DNA binding domain, results in conformational change of HSF2 that facilitates trimerization and DNA-binding [46]. SUMOylation of Oct4 at Lys118, which is located near the DNA binding domain, also causes conformational change of Oct4 that enhances its DNA binding activity [47]. Moreover, SUMOylation of signal transducer and activator of transcription-1 (STAT1) at Lys703 was found to enhance DNA-binding of STAT1 which further facilitates spatial memory formation in rats [11]. Furthermore, studies from NMR spectroscopy and protein-DNA cross-linking experiments reveal that the SUMO-1 molecule also possesses DNA-binding activity and SUMO-1 specifically binds to dsDNA without particular sequence [48]. It is conceivable that both the basic domain and the SUMO-1 molecule near the basic domain contribute to the DNA-binding activity of Hes-1. Whether the N-terminal domain of Hes-1 also confers a DNA-binding activity requires further investigation.

In this study we have found that GADD45α is a novel target of Hes-1. Hes-1 directly bound to the promoter of GADD45α and suppressed its promoter activity and gene expression. Sequence analysis indicated that, in addition to three N-boxes, there are also six E-boxes on the GADD45α promoter (nt. -867 ~ -862, nt. -852 ~ -849, nt. -773 ~ -768, nt. -771 ~ -766, nt. -716 ~ -711 and nt. -340 ~ -335). Therefore, Hes-1 may also suppress GADD45α expression through passive repression by preventing Mash1/E47 from binding to the E-box of GADD45α promoter. GADD45α transcripts can be rapidly induced by genotoxic stresses, and several transcription factors are involved in this process including p53, BRCA1, Oct1, NF-YA and WT1 [49-52]. In addition, GADD45α expression is reduced by c-Myc under various genotoxic stresses [53,54]. GADD45α expression could also be induced by H_2_O_2_ treatment [35], but the underlying mechanism is not known. In the present study, we have found that Hes-1 suppressed the expression of GADD45α under both normal condition and H_2_O_2_ stimulation. Furthermore, GADD45α expression is implicated in cell apoptosis. For example, overexpression of GADD45α was found to activate p38 MAPK and JNK and result in cell apoptosis [39]. UV radiation-induced apoptosis in keratinocytes was found decreased in GADD45α-deficient mice [38]. Consistent with these reports, here we found that overexpression of GADD45α enhanced, but knockdown of GADD45α decreased H_2_O_2_-induced apoptosis in HEK293T cells. These results together provide a novel protective mechanism of Hes-1 against H_2_O_2_-induced apoptosis through suppression of GADD45α expression. These results are congruent with the reports showing that Hes-1 plays a protective role against amyloid-beta-induced toxicity in neurons and that Hes-1 maintains stem cell survival [27,55]. Although there is report showing that GADD45α plays an anti-apoptotic role [40], this is probably due to different stimuli and cell types studied. Furthermore, we have found that Hes-1 siRNA at a concentration (20 nM) that did not affect cell survival alone greatly potentiated H_2_O_2_-induced cell death. But there are also reports showing that Hes-1 plays a pro-apoptotic role [56,57]. One possibility to explain this discrepancy is that the present study was carried out in HEK293T cells and the expression level of Notch receptor is very low in these cells, so the observed effects of Hes-1 on GADD45α expression and cell survival are likely unrelated to Notch signaling; instead, it serves as a general protective mechanism in various cell types.

On the other hand, we have also found that H_2_O_2_ from 0.01 mM to 0.5 mM produced a dose-dependent increase in Hes-1 SUMOylation but H_2_O_2_ decreased Hes-1 SUMOylation at higher doses (1 mM and 10 mM). Results from another study have shown that H_2_O_2_ at 100 mM produces a significant increase in global SUMOylation [22]. However, we did not examine Hes-1 SUMOylation by H_2_O_2_ at this concentration because most of the cells died under this concentration of H_2_O_2_ treatment. This is probably due to the difference between HeLa cells and HEK293T cells in terms of their resistance to H_2_O_2_ toxicity. In addition, Hes-1 SUMOylation may be more sensitive to the effect of H_2_O_2_ than global protein SUMOylation is. Furthermore, all the sumoylated forms of Hes-1 observed in the present study under H_2_O_2_ treatment are probably not included in global SUMOylation induced by H_2_O_2_ from that study because all sumoylated forms of Hes-1 are smaller than 72 kDa (Figure 1B and Figure 2A), whereas in that study all sumoylated proteins are larger than 100 kDa. Moreover, we have found that PIAS1 phosphorylation at Ser90 plays an important role in PIAS1 SUMOylation of Hes-1 in response to H_2_O_2_ stimulation. In speculation of the possible stress-activated kinases that phosphorylate PIAS1, IKKα and IKKβ could be the candidate kinases because H_2_O_2_ was shown to activate IKK activity [58] and IKKα was shown to phosphorylate PIAS1 at Ser90 to mediate anti-inflammation [59]. However, the involvement of other kinases can not be ruled out. For example, MAPK/ERK was shown to ameliorate H_2_O_2_ cytotoxicity in mouse kidney cells [60]. Whether MAPK/ERK also phosphorylates PIAS1 in response to H_2_O_2_ challenge requires further investigation. In addition, because Hes-1 SUMOylation down-regulated GADD45α expression, these results together suggest that H_2_O_2_-induced SUMOylation of Hes-1 may provide an endogenous protection mechanism against H_2_O_2_ insult.

In the present study, transfection of Hes-1 3KR did not completely block the suppressing effect of Hes-1 on GADD45α promoter activity and protein expression (Figure 6D and 6F). This is probably because that in addition to Hes-1 SUMOylation that affects Hes-1 binding to the GADD45α promoter, other posttranslational modifications of Hes-1 may also contribute to these observations. For example, we have previously found that the Hes-1 phosphorylation mutant, Hes-1S263A, decreases the stability and the transcriptional suppressing activity of Hes-1 [3]; presumably it would also decrease the amount of Hes-1 bound to DNA. Therefore, endogenous Hes-1 phosphorylation may also contribute to Hes-1 binding to the GADD45α promoter and regulate GADD45α expression even when normal Hes-1 SUMOylation was blocked. This speculation is supported by our findings that Hes-1 SUMOylation and Hes-1 phosphorylation are independent each other and that transfection of Hes-1 3KR did not completely block Hes-1 binding to the GADD45α promoter as determined by ChIP PCR. These results also suggest that there may be a synergistic effect of Hes-1 SUMOylation and Hes-1 phosphorylation on Hes-1 stabilization and Hes-1-mediated suppression of gene transcription. But these results do not exclude other possibilities that may also affect Hes-1 binding to the GADD45α promoter. Furthermore, the present results revealed that Hes-1 WT and Hes-1 3KR showed a similar ubiquitination level. These results indicated that Hes-1 SUMOylation stabilized the Hes-1 protein. Because protein SUMOylation was shown to affect the proteasomal degradation of protein, it is possible that Hes-1 SUMOylation may cause conformational change of Hes-1 that alters the interaction between Hes-1 and its E3 ubiquitin ligase. But other mechanisms may also involve in it. These results also do not exclude the possibilities that Hes-1 3KR may cause other changes because lysine is also subject to other post-translational modifications, such as acetylation.

In addition, unlike Hes-1 SUMOylation which was not previously reported in the literature, the cellular function of Hes-1 phosphorylation has been investigated. For example, Hes-1 was found phosphorylated by CaMKIIδ and CaMKIIδ activation of Hes-1 switches the function of Hes-1 from a repressor to an activator involved in neuronal differentiation [5]. Further, phosphorylation of Hes-1 by protein kinase C inhibits Hes-1 DNA-binding that is essential for neurite outgrowth induced by nerve growth factor in PC12 cells [4]. Whether Hes-1 SUMOylation is also involved in these cellular functions and, perhaps, other un-identified cellular functions requires further investigation.

The present results also demonstrated that overexpression of PIAS1 protected against H_2_O_2_-induced cell death, implicating that PIAS1 plays an anti-apoptotic role. These results are inconsistent with the reports showing that PIAS1 has a pro-apoptotic role [29,61]. One possibility to explain this discrepancy is perhaps due to different doses of PIAS1 used in these studies because only 0.2 μg PIAS1 plasmid DNA was transfected to HEK293T cells in the present study, but inducible PIAS1 expression was adopted in another study. We have similarly found that transfection of PIAS1 plasmid at a higher dose (1.6 μg) produced cell apoptosis. In addition, the role of PIAS1 in regulation of cell survival or apoptosis may also depend on the specific substrate that is sumoylated by PIAS1. In this study, we have found that co-transfection of the Hes-1WT plasmid enhanced the anti-apoptotic effect of PIAS1 on H_2_O_2_-induced cell death. This is probably because that more Hes-1 protein is available for PIAS1 SUMOylation of Hes-1 to take place, and Hes-1 SUMOylation plays an anti-apoptotic role. This explanation is supported by the observation that transfection of the Hes-1 sumo-mutant (Hes-1 3KR) prevented the anti-apoptotic effect of PIAS1. On the other hand, because PIAS1 inhibits STAT1 activity, the present results are congruent with the reports showing that STAT1 regulates cell death [62,63] and STAT1 mediates the neurotoxicity of amyloid-beta [64], although the apoptotic role of STAT1 may depend on the cell type and the specific STAT1 dimers formed [65,66].

## Conclusion

**Figure 9:**
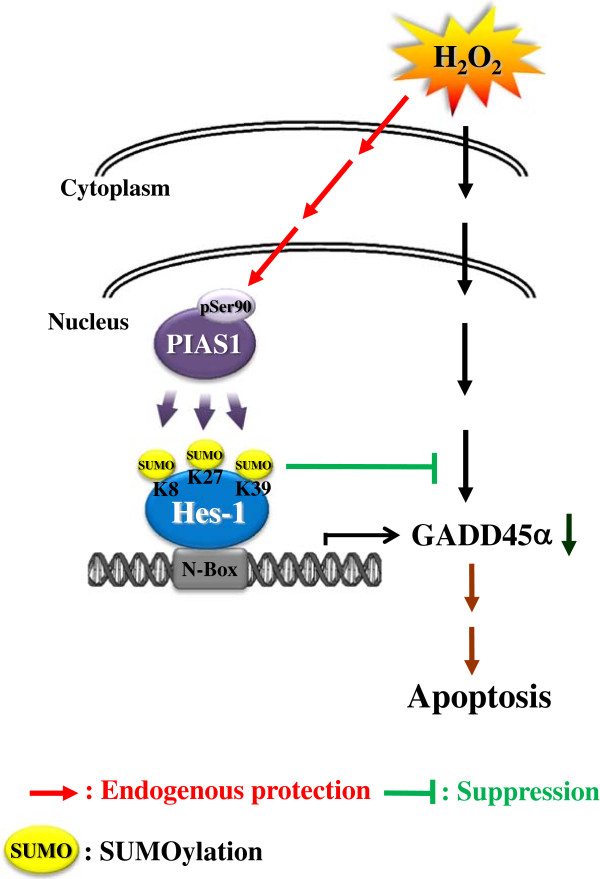
**A schematic diagram showing the relationship among PIAS1, Hes-1 SUMOylation and GADD45α expression that protects against H**_**2**_**O**_**2**_**-induced apoptosis.**

In conclusion, we have demonstrated that Hes-1 is a novel substrate of the SUMO E3 ligase PIAS1, and PIAS1 SUMOylation of Hes-1 stabilized Hes-1 and enhanced the transcriptional suppressing activity of Hes-1 on GADD45α expression. Furthermore, GADD45α increased cell apoptosis. Thus, Hes-1 SUMOylation by PIAS1 plays a protective role against cell apoptosis through enhanced suppression of GADD45α expression (Figure 9). These results provide the first evidence that posttranslational modification of Hes-1 by SUMOylation mediates cell survival.

## Competing interest

The authors declare that they have no competing interest.

## Author’s contributions

HYC and EHY designed the experiments and wrote the manuscript. HYC, SYL and CHL conducted the experiments and analyzed the data. All authors read related literatures, joined regular discussion and solved experimental problems. All authors read and approved this manuscript.
